# Increased Prothrombin, Apolipoprotein A-IV, and Haptoglobin in the Cerebrospinal Fluid of Patients with Huntington's Disease

**DOI:** 10.1371/journal.pone.0015809

**Published:** 2011-01-31

**Authors:** Yen-Chu Huang, Yih-Ru Wu, Mu-Yun Tseng, Yi-Chun Chen, Sen-Yung Hsieh, Chiung-Mei Chen

**Affiliations:** 1 Department of Neurology, Chang Gung Memorial Hospital, Chang-Gung University College of Medicine, Taipei, Taiwan, People's Republic of China; 2 Clinical Proteomics Center, Chang Gung Memorial Hospital, Taipei, Taiwan, People's Republic of China; Case Western Reserve University, United States of America

## Abstract

Huntington's disease (HD) is a progressive neurodegenerative disease caused by an unstable CAG trinucleotide repeat expansion. The need for biomarkers of onset and progression in HD is imperative, since currently reliable outcome measures are lacking. We used two-dimensional electrophoresis and mass spectrometry to analyze the proteome profiles in cerebrospinal fluid (CSF) of 6 pairs of HD patients and controls. Prothrombin, apolipoprotein A-IV (Apo A-IV) and haptoglobin were elevated in CSF of the HD patients in comparison with the controls. We used western blot as a semi-quantified measurement for prothrombin and Apo A-IV, as well as enzyme linked immunosorbent assay (ELISA) for measurement of haptoglobin, in 9 HD patients and 9 controls. The albumin quotient (Qalb), a marker of blood-brain barrier (BBB) function, was not different between the HD patients and the controls. The ratios of CSF prothrombin/albumin (prothrombin/Alb) and Apo A-IV/albumin (Apo A-IV/Alb), and haptoglobin level were significantly elevated in HD. The ratio of CSF prothrombin/Alb significantly correlated with the disease severity assessed by Unified Huntington's Disease Rating Scale (UHDRS). The results implicate that increased CSF prothrombin, Apo A-IV, and haptoglobin may be involved in pathogenesis of HD and may serve as potential biomarkers for HD.

## Introduction

Huntington's disease (HD) is a progressive neurodegenerative disease with autosomal dominant inheritance, characterized by cognitive decline, movement disorder and different psychiatric manifestations. It is caused by an unstable CAG trinucleotide repeat expansion in the gene encoding huntingtin protein [1] resulting in neuronal dysfunction and death predominantly in the striatum and cortex [2]. Several pathogenic mechanisms have been identified, including neuronal aggregation of the mutated protein [3], transcriptional dysregulation [4], excitotoxicity [5], altered energy metabolism [6], [7], mitochondrial dysfunction [8], [9], impaired axonal transport, and altered synaptic transmission [10], [11].

Therapeutic strategies that prevent HD progression are not available yet. While many potential beneficial treatments have been reported in cell or/and animal models, reliable pre-symptomatic/symptomatic biomarkers that can indicate the disease status and test the therapeutic efficacy are lacking. Because cerebrospinal fluid (CSF) is located around and inside the brain, biomarkers originating from it may provide insights into the disease pathophysiology and severity as well as monitoring responses to the preventive, disease-modifying, and symptomatic therapies.

Fang and colleagues reported an extensive list of proteins identified in HD CSF relative to control using current proteomics technology, supporting the idea of CSF as a rich source of biomarkers [12]. In several reports, plausible evidence supports an increased activity of transglutaminases in HD that generates elevated levels of γ-glutamyl-ε-lysine cross-links and γ-glutamylpolyamine residues in CSF [13], [14]. However, the γ-glutamyl-ε-lysine cross-links are also elevated in Alzheimer's disease (AD) and vascular dementia [15], indicating that increased transglutaminase activity is a common phenomenon in a variety of neurodegenerative disease and not specifically for HD [16]. In another study, F2-isoprostane, a marker of oxidative stress, was increased in CSF from HD patients, but the level was not correlated with disease duration [17]. Cocaine- and amphetamine-regulated transcript (CART) was also elevated in CSF of HD patients, probably reflecting the hypothalamic lesion in regulating energy homeostasis and emotion, but the level was not correlated to CAG repeat length, body mass index, or other clinical features [18]. CSF neurofilament was increased in HD subjects and was correlated with scores on the Unified Huntington's Disease Rating Scale (UHDRS) total functional capacity [19]. Clusterin, inteleukin 6 (IL-6), and interleukin 8 (IL-8) in both serum and CSF were up-regulated and correlated to disease severity, indicating a neuroinflammatory activation in HD [20], [21]. Despite an extensive list of CSF proteins identified in HD by Fang and colleagues [12], a subsequent study has not been conducted yet to further verify the differentially-expressed protein molecules as reliable biomarkers.

Given that valid CSF biomarkers that are specific for HD and correlated with disease severity are imperative, we set out to use clinical proteomics to investigate the CSF protein profile of HD patients, which may be helpful to shed light on the pathophysiology of HD and to identify potential biomarkers.

## Materials and Methods

### CSF specimen and preparation

CSF and serum samples were collected from 9 HD (5 males, 4 females, Table 1) patients who were recruited from neurological clinic of Chang Gung Memorial Hospital and genetically diagnosed. The UHDRS of each patient was assessed by clinicians (Yih-Ru Wu and Chiung-Mei Chen) in outpatient clinics before the CSF and serum were collected and investigated for proteomic profile. Therefore, the clinicians who assessed the UHDRS did not know the CSF results before scoring. The proteomic studies of CSF samples were subsequently performed by a different person, who was blinded to the identity and the disease severity of each patient. CSF and serum samples from 18 controls (7 males, 11 females, Table 1) were collected from patients with other neurological diseases who were admitted due to migraine, tension headache, spontaneous intracranial hypotension, amyotrophic lateral sclerosis, and degenerative spine diseases. These control patients had no systemic infection; chronic renal, cardiac or liver dysfunction; autoimmune diseases; or malignancies. The 18 controls were divided into 4 groups with one for two-dimensional electrophoresis (2-DE) and the other overlapping three for quantification of prothrombin, Apo-A IV, and haptoglobin. Body mass index (BMI) was calculated by weight in kilograms divided by squared height in meters for each individual. The examination of the control and HD CSF samples also showed an absence of inflammation (no pleocytosis). Venous blood was obtained simultaneously with the CSF sampling for each subject. White blood cell counts, and albumin concentration of the CSF and serum were analyzed automatically using Synchron LX®20 PRO (Beckman Coulter, Brea, California, USA) by the Department of Clinical Pathology, Chang Gung Memorial Hospital. The albumin quotient (Qalb) was derived from CSF albumin/serum albumin, where the CSF or serum albumin was an absolute concentration. This study was performed under a protocol approved by the institutional review boards of Chang Gung Memorial Hospital (ethical license No: 98-0125C) and all examinations were performed after obtaining written informed consents.

**Table 1:** Clinical characteristics of the Huntington's disease (HD) patients and the controls.

	Controls (n = 18)	HD patients (n = 9)
Gender (male/female)	7/11	5/4
Age (years)	45.8±3.0 (26–64)#	43.4±3.4 (25–57)
Age at symptom onset (years)		38.9±4.0 (15–53)
Body mass index	23.4±0.8	21.0±1.1*
Disease duration (years)		4.9±1.0 (2–10)
Expanded CAG repeat No		46.1±2.3 (41–62)
UHDRS		
Motor score		32.2±7.4 (9–69)
Independence scale		78.9±6.6 (40–100)
Functional capacity		9.2±1.2 (3–13)

Values are expressed as means ± SE (range; minimum–maximum).

UHDRS: The Unified Huntington's Disease Rating Scale. Scale ranges (normal to most severe) include motor score (0 to 124), independence score (100 to 10), and functional capacity (13 to 0).

#Three different groups from controls (9 in each group) were compared with 9 HD patients for prothrombin/Alb, Apo A-IV/Alb, and haptoglobin, respectively (The ages of the 3 groups were 41.7±4.3, 45.7±4.7, and 41.1±4.1).

*P = 0.09.

CSF samples were centrifuged immediately after collecting to eliminate cells and other insoluble material, aliquoted and stored at −80°C until analysis. Blood samples were kept at 4°C for 1 h and then centrifuged. The serum was aliquoted and stored at −80°C until analysis.

### UHDRS

UHDRS [22] including motor score, independence scale, and functional capacity, was used to assess the severity of HD (Table 1). Worse severity of HD scores higher in motor score and lower in independence scale and functional score.

### 2-DE

CSF samples were cleaned up by ReadyPrep 2-DE Clean-Up Kit (Bio-Rad, Herculus, CA) following the manufacturer's instruction. Briefly, each CSF sample was first mixed with 3-fold volume of precipitation agent 1 and kept on ice for 15 min, and then 3-fold sample volume of precipitation agent 2 was added. The mixture was centrifuged at 19,000×*g* for 5 min. The pellet was washed by wash reagent 1 and centrifuged again. After the pellet was re-suspended with distilled water, pre-chilled wash reagent 2 and wash reagent 2 additive were added. The mixture was incubated at −20°C for 30 min and vortexed every 10 min followed by a centrifugation at 19,000×*g* for 5 min. The protein pellets were solubilized in rehydration buffer (85 mM DTT, 1.4% biolyte pH 3–10, 4.4 M urea, 1.8 M thiourea, 3.5% CHAPS).

Each sample containing 350 µg of protein was diluted to 330 µl with rehydration buffer and kept at room temperature for 2 h. The isoelectric focusing electrophoresis (IEF) was performed with pH 3–10 nonlinear immobilized pH gradient (IPG) strips (Bio-Rad, Herculus, CA) at 18°C as the following steps: active rehydration at 50 V for 16 h, then fast to 300 V for 1h, slow to 3500 V for 3h, kept at 3500 V for 3h, finally fast to 8000 V until achieved 63000 Vh. After IEF, the strip was equilibrated with equilibration buffer (6 M urea, 30% glycerol, 2% SDS) containing 2% DTT for 15 min at room temperature, and then incubated with 2.5% IAA in equilibration buffer for another 15 min. The secondary dimensional separation was performed using 11.5% SDS polyacrylamide gel electrophoresis (SDS-PAGE).

### Protein visualization and image analysis

The 2-DE gels were fixed with 7% acetic acid and 10% methanol for 60 min followed by staining in a SYPRO Ruby solution (Molecular Probes, Eugene, OR) for 6 h. After that, gels were washed for 10 h with 7% acetic acid and 10% methanol to remove the background, and then stored in distilled water at 4°C. Gels were scanned by a ProXPRESS 2D Proteomic Imaging System (PerkinElmer, MA, USA) at excitation/emission values of 460 nm/650 nm and resolution of 100 µm. Gel images were analyzed by Progenesis software (Nonlinear Dynamics, Durham, NC) and a cut-off value 1.5-fold change of spot normalized volume was used to be an estimation of significant difference.

### In-gel digestion

The interesting spots were cut from 2-DE gel. The gel particles were incubated in 25 mM NH_4_HCO_3_ buffer containing 55 mM DTT, pH 8.5, for 1 h at 37°C and then incubated in 25 mM NH_4_HCO_3_ containing 100 mM IAA, pH 8.5, for 1 h at 37°C in dark. After washing twice with 25 mM NH_4_HCO_3_/50% acetonitrile for 15 min, gel particles were dehydrated with acetonitrile followed by rehydration with trypsin solution (5 µl of 25 mM NH_4_HCO_3_, pH 8.5, containing 20 ng trypsin) and then incubated at 37°C for 16 h. The peptides were extracted by 5% TFA in 50% acetonitrile and analysis by mass spectrometry.

### Mass spectrometry (MS) analysis and protein identification

Matrix-assisted laser desorption/ionization time-of-flight mass spectrometry (MALDI-TOF MS) analyses were performed using an Ultraflex MALDI-TOF MS instrument (Bruker Daltonics). Samples were premixed with matrix solution (1 mg/ml α-Cyano-4-hydroxycinnamic acid (CHCA) in 70% acetonitrile, 0.1% v/v TFA) at 1∶1 ratio and spotted onto the AnchorChip. A nitrogen laser (wave length, 337 nm) operating at 20 Hz was used for ionization. For matrix suppression, a gating factor with signal suppression up to 800 Da was performed. A calibration mixture of known peptides/proteins in a mass range of 1,000∼25,000 Da was set for mass calibration. To increase detection sensitivity, excess matrix was removed with ten shots at a laser power of 60% before data acquisition. Fifty laser shots at three different spot positions were acquired in each spectrum. All of the peaks with S/N>5 in a mass range of 1,000∼25,000 Da were recorded using the AutoXecute tool of the flexControl acquisition software (Version 2.0, Bruker Daltonics).

Matrix-assisted laser desorption/ionization time-of-flight tandem mass spectrometry (MALDI-TOF MS/MS) analyses were performed by a dedicated Q-Tof Ultima™ MALDI instrument (Micromass, Manchester, UK) and the systems were operated under MassLynx 4.0 and raw MS data were processed for database searching using ProteinLynx Global Server 2.0. Each sample was premixed 1∶1 with matrix solution (5 mg/ml CHCA in 50% acetonitrile, 0.1% v/v TFA and 2% w/v ammonium citrate) and spotted onto the 96 well formats MALDI sample stage. Within each well, peaks of parent ions which were within the *m/z* 800∼3,000 range with intensity above 10 counts ± include/exclude list will be selected automatically for collision-induced dissociation (CID) MS/MS using argon as collision gas and a mass dependent ±5V rolling collision energy until end of probe pattern was reached, starting from the most intense peak. The low mass and high mass resolution of the quadrupole were both set at 10 to give a precursor selection window of about 4 Da wide. The instrument was externally calibrated to less than 5 ppm accuracy over the mass range of *m/z* 800∼3,000. At a laser firing rate of 10 Hz, individual spectra from 5 second integration period acquired for each of the MS survey and MS/MS performed were combined, smoothed, deisotoped (fast option), and centroided using the Micromass ProteinLynx™ Global Server (PGS) 2.0 data processing software. The combined PMF and MS/MS ion meta data were searched against the specified protein database within the PGS 2.0 workflow.

The resulting peptide data from both mass and tandem mass were searched against SWISS-PROT database using MASCOT software on the matrixscience website (www.matrixscience.com). Matching peptides with one missed cleavage were considered as adequacy. For MS data searching, a mass deviation of 50 and 75 ppm was allowed. For MS/MS ion searching, a 50 ppm peptide mass tolerance and 0.6 Da ms/ms tolerance was applied.

### Western blot analysis

Equal amount protein of each CSF or serum sample from 9 HD patients and 9 controls was mixed with sample buffer (Invitrogen, Carlsbad, CA) and reducing agent (Invitrogen, Carlsbad, CA). Each sample was heated at 70°C for 10 min, and then separated by NuPAGE Novex Bis-Tris 4–12% gel (Invitrogen, Carlsbad, CA) and transferred to polyvinylidene fluoride (PVDF) membranes (Millipore, Billerica, MA). After overnight blocking, the membranes were incubated with 1 µg/ml anti-prothrombin rabbit polyclonal antibody (Abnova, Taipei, Taiwan) or anti-prothrombin mouse polyclonal antibody (Abnova, Taipei, Taiwan) at 1∶ 1000 dilution, anti-apolipoprotein A-IV (Apo-A IV) polyclonal antibody (Abnova, Taipei, Taiwan) at 1∶2000 dilution in Tris buffer saline containing 0.05% Tween 20 (1× TBST) for 3 h, and anti-albumin antibody (Sigma, Saint Louis, MO) at 1∶200,000 dilution in 1× TBST for 2 h. The membranes were washed and incubated in 1× TBST containing horseradish peroxidase conjugated goat anti-mouse IgG secondary antibody or donkey anti-rabbit IgG secondary antibody (Pierce, Rockford, IL) for 2 h. For membranes stained with mouse anti-prothrombin and anti-Apo A-IV primary antibody, 0.04 µg/ml secondary antibody was applied; for membranes stained with rabbit anti-prothrombin primary antibody, 0.013 µg/ml secondary antibody was applied; for membranes stained with anti-albumin primary antibody, 0.002 µg/ml secondary antibody was added. Immuno-reactive bands were visualized with SuperSignal West Pico Chemiluminescent Substrate (Pierce, Rockford, IL) and detected with Kodak BioMax light film. The resulting bands were scanned and measured for density using Image Pro software (Image Pro Plus 5.0, Media Cybernetics, MD). Western blot experiments for both Apo A-IV and prothrombin were performed in triplicate to reduce experimental error and the mean was used in statistical analysis. The level of prothrombin and Apo A-IV in CSF or serum was expressed as the ratio of prothrombin and Apo A-IV over albumin density in each sample on the western blot (prothrombin/Alb and Apo A-IV/Alb, respectively).

### Enzyme linked immunosorbent assay (ELISA) for haptoglobin

CSF and serum haptoglobin levels were assessed by AssayMax ELISA Kit (Assaypro, Winfield, MO, USA) according to the manufacturer's instruction.

### Statistical analysis

All statistical analyses were performed using Statistical Program for Social Sciences (SPSS) statistical software (version 16, Chicago, IL, USA). For each set of values, data were expressed as mean ± standard error (SE) or median (interquartile range) where appropriate. Differences in spot intensities in 2-DE gels between HD patients and controls were analyzed by the paired Student's *t*-test. Mann-Whitney U test was used to examine the differences between HD patients and controls in BMI, prothrombin/Alb, Apo A-IV/Alb, and haptoglobin concentration in CSF and serum. Spearman correlation was used to test if the examined protein levels were correlated with age. Mann-Whitney U test was used to examine if the protein levels were affected by gender. Linear regression analysis was applied to evaluate the correlation of each subscale in UHDRS to Apo A-IV/Alb, prothrombin/Alb, and haptoglobin concentration in CSF, respectively. The correlation of BMI with Apo A-IV/Alb in CSF of HD patients was also analyzed by linear regression. A *p*<0.05 was considered statistically significant. The present sample sizes achieve a power of 71%, 94%, and 80% for haptoglobin, Apo A-IV/Alb, and prothrombin/Alb, respectively, to detect differences between the mean of control group and that of case group with the estimated standard deviations at the level of 0.05.

## Results

### CSF proteome profile in HD patients and controls

**Figure 1:**
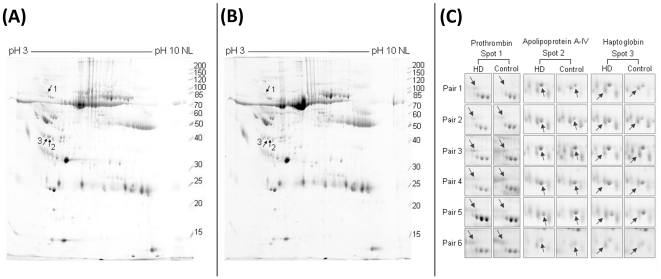
2-DE maps of CSF proteomes. Representative 2-DE maps of CSF proteome from a patient with HD (A) and a control (B) are shown. Spots 1–3 indicate proteins with up-regulation in HD. Cropped images containing spots corresponding to prothrombin (spot 1), apolipoprotein A-IV (Apo A-IV, spots 2) and haptoglobin (spot 3) (indicated by arrowheads) derived from 2-DE maps of six CSF samples of patients with HD and paired control samples (C).

We investigated the proteome profile in CSF of 6 HD patients (age, 42±4.8) and 6 age-matched controls (age, 44.4±3.21) using gel-based proteomics technology. The disease and control samples were aligned randomly to get pairs for 2-DE gel analysis. A representative pair of resulting 2-DE gels was shown in figure 1. There were at least three protein spots (spots 1–3) expressed at higher level on the gel of CSF from the HD patient (Fig. 1A) compared to the corresponding control (Fig. 1B). Although some other protein spots expressed at differential level between HD and control CSF, we only showed the protein spots whose volume after normalization (spot volume over total spots volume on the gel, analyzed by Progenesis software) have at least 1.5-fold change in at least four pairs of specimens, or *P*<0.05 analyzed by paired *t*-test. MALDI-TOF MS and MALDI-TOF MS/MS were applied to identify the proteins corresponding to these three spots (Table 2). The three up-regulated proteins in HD CSF were prothrombin, Apo A-IV, and haptoglobin. The cropped images of these three spots from 2-DE maps of six sample pairs are shown in Figure 1C.

**Table 2:** Differentially-expressed protein molecules in CSF of Huntington's disease (HD) patients.

Up-regulated proteins in CSF of patients with HD identified by MALDI-TOF MS							
Spota)	Accession No.b)	Protein name	pI/M.W.	Scorec)	Coveraged) (%)	Mean of fold changese) (HD/C)	Frequencyf), *P* value
1	P00734	Prothrombin	5.64/69209	82	23	1.66	5/6, *P* = 0.03
2	P06727	Apolipoprotein A-IV	5.28/45371	174	42	1.67	4/6, *P* = 0.04

Up-regulated proteins in CSF of patients with HD identified by MALDI-TOF MS/MS							
Spota)	Accession No.b)	Protein name	Parent peak mass	Sequence	Scorec)	Mean of fold changese) (HD/C)	Frequencyf), *P* value
3	P00783	Haptoglobin	1722.7991	K.YVMLPVADQDQCIR.H	51	1.96	5/6, *P* = 0.07

a) Spots numbered as shown in Figure 1.

b) The accession numbers on SWISS-PROT database.

c) The MASCOT search score of identified proteins.

d) The percentage of sequence coverage of matched peptides in the identified protein.

e) The mean protein expression level in the HD patients over that in the controls in Figure 1C.

f) The frequency of the target protein, for which the expression ratio of HD/C was >1.5. *P*<0.05 indicates a significant difference in expression levels between HD patients and controls analyzed by paired student's *t*-test.

The expression level of prothrombin, spot 1 in Fig. 1, was 1.66-fold higher in HD versus control samples, which was found in five of six comparisons (*p* = 0.003). Another up-regulated protein, Apo A-IV (spot 2 in Fig. 1) was 1.67-fold increased in CSF of the HD patients, and the Apo A-IV level was higher in four HD samples compared with the controls (*p* = 0.004). The haptoglobin (spot 3 in Fig. 1) level revealed a 1.96-fold increase in HD group compared with the controls, which was found in five of six comparisons (*p* = 0.007).

### Increased prothrombin, Apo A-IV and haptoglobin levels in CSF of HD patients

**Figure 2:**
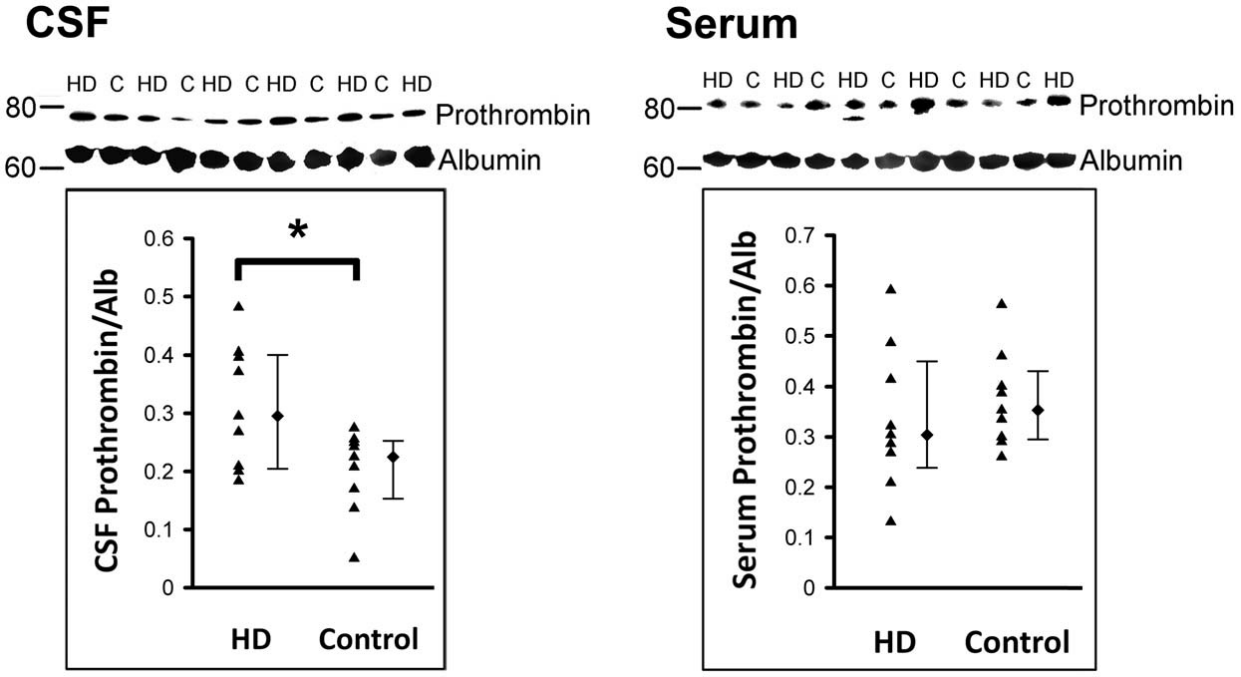
Representative Western blots of prothrombin (72 kD) and albumin (64 kD) in CSF and serum of HD patients and their corresponding controls (C). The resulting data are illustrated in scatter plots with median and interquartile range. The prothrombin/Alb ratio in CSF of HD patients (n = 9) is higher than that in controls (n = 9). *p<0.05, Mann-Whitney U test.

The increased levels of CSF prothrombin, Apo A-IV, and haptoglobin were further examined in 9 HD patients compared with 9 age-matched controls using western blot analysis or ELISA. The levels of prothrombin in CSF and serum from 9 HD patients (age, 43.4±3.4) and 9 age-matched controls (age, 41.7±4.3) are shown in Fig. 2. Age did not has an effect on CSF prothrombin/Alb (*p* = 0.90), Apo A-IV/Alb (*p* = 0.30), and haptoglobin (*p* = 0.65) levels. The CSF prothrombin/Alb ratio (male, 0.30 [0.24 to 0.40]; female, 0.21 [0.17 to 0.27], *p* = 0.05), Apo A-IV/Alb ratio (male, 0.12 [0.05 to 0.17]; female, 0.07 [0.06 to 0.10], *p* = 0.16), and haptoglobin levels (male, 0.9 [0.24 to 2.67]; female, 0.42 [0.23 to 0.82], *p* = 0.18) showed no significant difference between male and female group. The prothrombin/Alb ratio in CSF of HD patients (0.30 [0.21 to 0.40]) was increased compared with the controls (0.22 [0.16 to 0.26]) (*p* = 0.048). The ratio of prothrombin/Alb in serum of HD patients (0.30 [0.24 to 0.45]) was not different from those of controls (0.35 [0.30 to 0.43]). Albumin quotient (Qalb, CSF albumin/serum albumin), a marker of blood-brain barrier (BBB) function revealed no significant difference when comparing the HD patients (4.7 [4.1 to 6.6] *10^−3^) with the controls (4.0 [3.4 to 6.3]*10^−3^).

**Figure 3:**
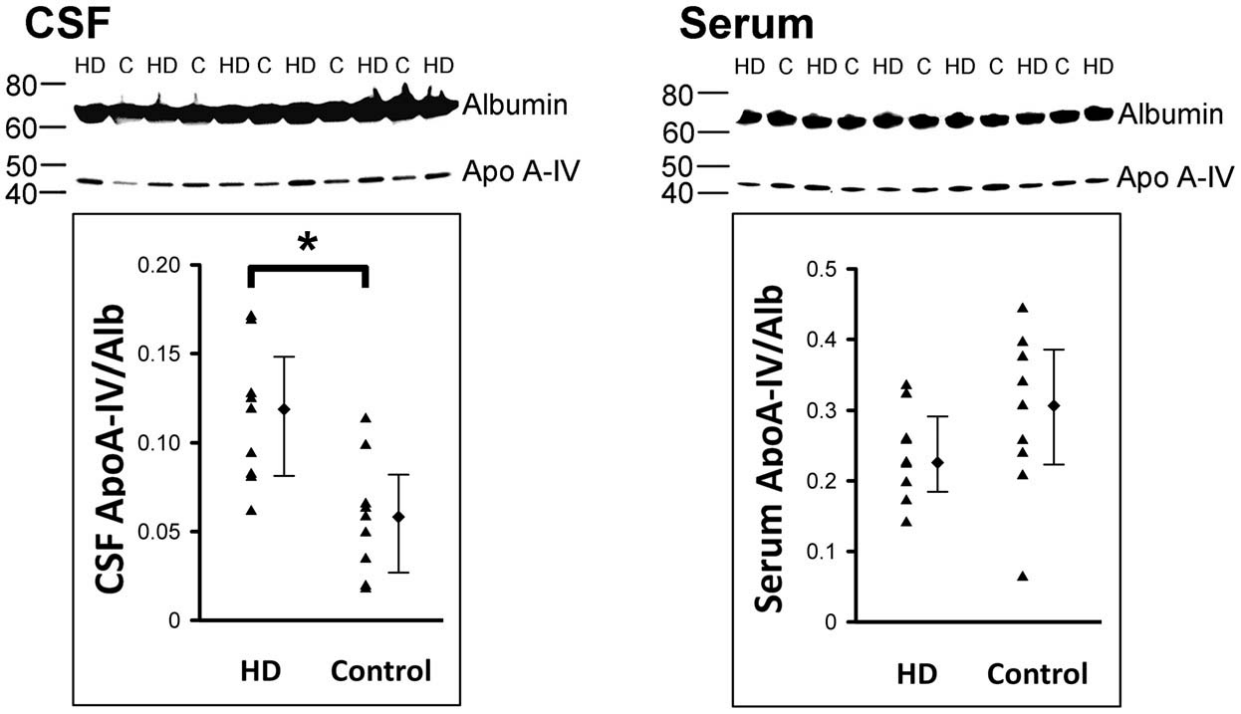
Representative Western blots of Apo A-IV (46 kD) and albumin (64 kD) in CSF and serum, respectively, of HD patients and their corresponding controls (C). The Apo A-IV/Alb ratios in CSF and serum in comparison with their corresponding control groups are illustrated in scatter plots with median and interquartile rang, respectively. The Apo A-IV/Alb ratio in CSF of HD patients (n = 9) is significantly higher when compared with the controls (n = 9). *p<0.01, Mann-Whitney U test.

The Apo A-IV/Alb ratios in CSF and serum from 9 HD patients (age, 43.4±3.4) and 9 age-matched controls (age, 45.7±4.7) are shown in Fig. 3. The ratio of Apo A-IV/Alb in CSF of HD patients (0.12 [0.08 to 0.15]) was significantly higher than that of controls (0.06 [0.03 to 0.09]) (*p* = 0.005), while ratio of Apo A-IV/Alb in serum of HD patients (0.23 [0.19 to 0.29]) was not different from those of controls (0.31 [0.23 to 0.39]). The Qalb were not different between HD patients (4.7 [4.1 to 6.6]*10^−3^) and controls (3.4 [2.6 to 6.6]*10^−3^). CSF Apo A-IV/Alb ratio was not correlated to BMI in HD patients. In order to know if the elevated CSF Apo A-IV is specific to HD, we compared CSF Apo A-IV/Alb ratios between 5 controls (0.058 [0.013 to 0.154]) and 5 patients with Guillain–Barré syndrome (0.095 [0.055 to 0.126]) and there was no difference between them.

**Figure 4:**
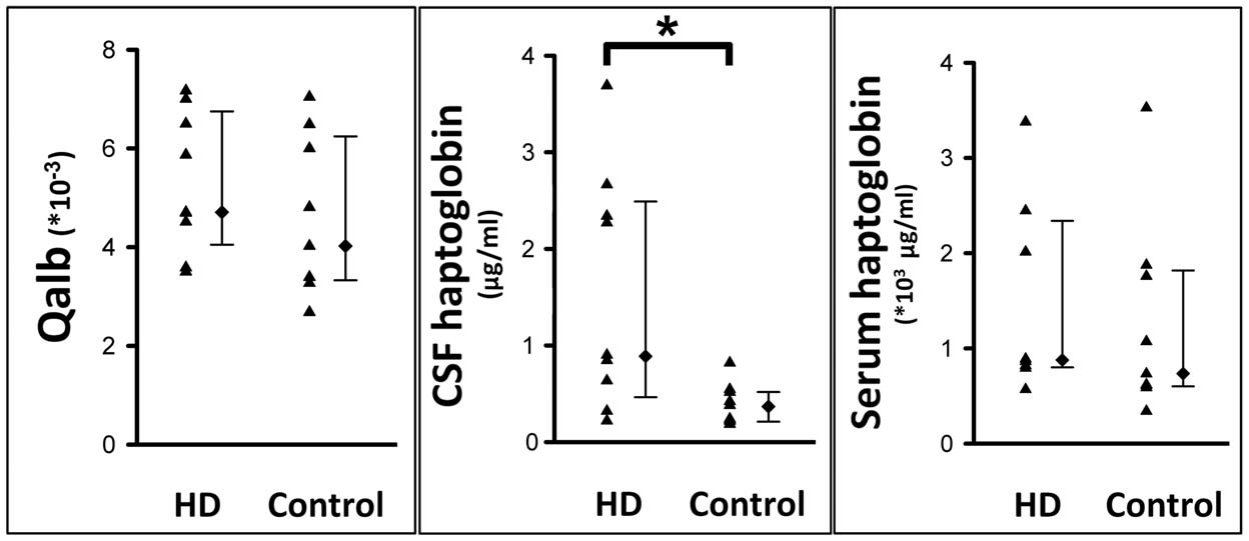
Qalb, CSF haptoglobin, and serum haptoglobin concentration in HD patients. Data are illustrated in scatter plots with median and interquartile rang. Either the Qalb or serum haptoglobin concentration was not different between HD (n = 9) and the controls (n = 9), whereas the CSF haptoglobin was significantly increased in HD. *p<0.05, Mann-Whitney U test.

The haptoglobin levels in CSF and serum from 9 HD patients (age, 43.4±3.4) and 9 age-matched controls (age, 41.1±4.1) are shown in Fig. 4. CSF haptoglobin concentration of HD patients (0.9 [0.48 to 2.50] µg/ml) was significantly higher than those of controls (0.38 [0.22 to 0.53] µg/ml) (*p* = 0.014), whereas haptoglobin concentration in serum of HD patients (876 [799 to 2338] µg/ml) was not different from those of controls (902 [612 to 1846] µg/ml). The Qalb was not different between HD patients (4.7 [4.1 to 6.8]*10^−3^) and controls (4.0 [3.4 to 6.3]*10^−3^).

### The correlation of prothrombin/Alb, Apo A-IV/Alb, and haptoglobin concentration in CSF to UHDRS subscales, respectively

**Figure 5:**
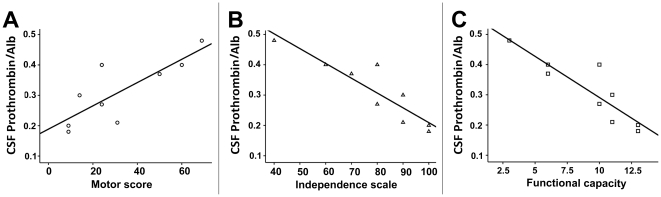
Linear regression of prothrombin/Alb to motor score, independence scale, and function capacity of UHDRS, respectively. The ratio of prothrombin/Alb was significantly correlated to subscales of UHDRS, with a positive correlation to motor score (A, *r* = 0.810, *p* = 0.008) and a negative correlation to independence scale (B, *r* = −0.908, *p*<0.001) and functional capacity (C, *r* = −0.891, *p* = 0.001).

The ratio of CSF prothrombin/Alb was significantly correlated to subscales of UHDRS, with a positive correlation to motor score (*r* = 0.810, *p* = 0.008) and a negative correlation to independence scale (*r* = −0.908, *p*<0.001) and functional capacity (*r* = −0.891, *p* = 0.001), respectively (Fig. 5). However, there was no significant correlation of the CSF Apo A-IV/Alb ratio to motor score (*r* = 0.394, *p* = 0.294), independence scale (*r* = −0.447, *p* = 0.228) and functional capacity (*r* = −0.521, *p* = 0. 151), as well as the CSF haptoglobin concentration to motor score (*r* = −0.047, *p* = 0. 905), independence scale (*r* = 0.067, *p* = 0.864) and functional capacity (*r* = 0.002, *p* = 0.995). There was no significant correlation of the CAG repeat numbers to CSF Apo A-IV/Alb ratio (r = 0.539, p = 0.134), CSF prothrombin/Alb (r = 0.644, p = 0.061) and CSF haptoglobin concentration (r = 0.008, p = 0.944).

## Discussion

In this study, using 2-DE and MS analysis we have identified three up-regulated protein molecules, prothrombin, Apo A-IV, and haptoglobin, in CSF of HD patients in comparison with controls. We have further used western blot analysis to measure prothrombin and Apo A-IV levels and ELISA to measure haptoglobin levels. The CSF prothrombin/Alb and Apo A-IV/Alb ratios and haptoglobin concentration in HD patients were increased, respectively, when compared with the controls. Importantly, the CSF prothrombin/Alb ratio was significantly correlated to the disease severity indicated by motor, independence, and functional score of UHDRS.

Prothrombin is synthesized in the liver as a precursor of thrombin. It is cleaved into kringle-1 and kringle-2 domain and active thrombin by prothrombinase [23], which is a crucial step in the coagulation cascade [24]. Both prothrombin and thrombin have been detected locally in the brain and may exert physiological and pathological functions [25], [26]. Thrombin can signal via G protein-coupled protease-activated receptors (PARs) [27] and has been considered as a potent mitogenic and proinflammatory agent to microglia and astrocytes [28], [29], thereby regulating neuronal function. Prothrombin kringle-2 domain participates in cortical neuron death through microglial NADPH oxidase-mediated oxidative stress [30]. Prothrombin and thrombin probably play important roles in the inflammatory process through the activation of PAR and microglia. Some neurodegerative diseases are characterized by increased levels of both active thrombin and PAR-1. Parkinson's disease (PD) is characterized by the selective loss of dopaminergic neurons in the substantia nigra as well as the activation of microglia [31]. The selected dopaminergic neuronal death has been shown as a result of microglia activation by thrombin [32], [33]. In AD, thrombin and prothrombin are expressed in neurons and glial cells and accumulated in senile plaques, reactive microglial cells, and neurofibrillary tangles in brains [26]. In HD, aggregates of mutant huntingtin exist in neurons, astrocytes, and microglia, resulting in the altered neuronal-glial interactions [34]. The mutant huntingtin may enhance the ability of microglia to produce proinflammatory mediators (including IL-6, IL-8, and TNF-α) and contribute to neurodegeneration in HD [20], [34]. Since prothrombin was thought to be involved in the inflammatory process through the activation of PAR and microglia in the central nervous system (CNS), the increased CSF prothrombin hints the inflammatory process in HD. However, the mechanism of how prothrombin is increased in CSF and how it is involved in the pathogenesis of HD remain to be determined. It is worth to note that ratio of CSF prothrombin/Alb was significantly proportional to the disease severity, which may provide a potential biomarker indicating the disease status. Since we did not examine if CSF prothrombin/Alb ratio of other diseases are increased, whether the elevated prothrombin is specific to HD remains to be clarified.

Apo A-IV is a glycoprotein synthesized mainly by the human intestine and also in hypothalamus. Dense Apo A-IV staining has been shown in the arcuate (ARC) hypothalamic nuclei, where Apo A-IV was co-localized with pro-opiomelanocortin (POMC, a body weight decreasing peptide), suggesting that the brain Apo A-IV suppresses food intake probably by stimulating the ARC POMC system [35]. Its intestinal and hypothalamic syntheses are markedly stimulated by fat absorption. Apo A-IV could be up-regulated in hypothalamus by leptin that acts tonically as an afferent signal from adipose tissue to the brain, as part of a negative feedback loop to regulate energy balance [36]. The elevated level of Apo A-IV in CNS subsequently inhibits food intake and regulates the long-term balance of body weight [37], [38]. Increased Apo A-IV has been found in CSF of HD patients by Fang et al. and its origin speculated by the authors may be from peripheral blood [12]. However, a recent study suggested that the circulating Apo A-IV in blood is unable to cross the blood brain barrier (BBB) [36]. In contrast to the proposal presented by Fang et al., our study shows that serum Apo A-IV is not increased and Qalb in HD is not different from that in control, indicating intact BBB, both of which suggest that the increased CSF Apo A-IV may largely originate from the CNS rather than from peripheral blood, although some proteins may be delivered into CNS by specific transporters. In HD, weight loss is a characteristic feature in advanced as well as early stage [7], but the mechanism is not clearly known. A serum metabolic profile indicative of a pro-catabolic phenotype in early HD preceding symptom onset has been shown in both HD patients and transgenic mice [6]. Increased CART, a strong anorectic peptide involved in regulating satiety and body weight, has been shown in CSF of HD patients, also indicating a negative energy balance in HD [18]. The pro-catabolic state in HD patients may account for the cachexia-like features seen in the late disease stage and may also account for the lower than expected weight of patients in the early disease stage. Since Apo A-IV produced in CNS may exert its anorectic action, the finding of increased CSF Apo A-IV in present study may implicate an altered energy metabolism, which may be related with the weight loss in HD patients. It is worth to note that the BMI of HD patient is lower than that of the controls, although it did not reach a statistical significance. The CSF Apo A-IV level, however, was not correlated with BMI in HD patients, which suggests that BMI may be affected by other factors than CNS Apo A-IV only. Further HD animal studies are warranted to explore the mechanism of how CSF Apo A-IV is increased and if it correlates to weight loss in HD. Although the increased CSF Apo A-IV was not found in patients with Guillain–Barré syndrome, since the number of the patients is small, further studies of a large series of samples with other neurological diseases will be warranted to verify whether the elevated Apo A-IV is specific to HD.

Haptoglobin, synthesized primarily by hepatocytes, is stimulated by infection or inflammation. It can bind irreversibly to oxygenated and free hemoglobin (Hb) and thereby plays an antioxidative activity [39]. The antioxidative effect is also observed in CNS. It was found to be produced by oligodendrocytes and play a protective role against Hb-mediated toxicity in intracerebral hemorrhage [40]. Furthermore, haptoglobin was found to be synthesized in reactive astrocytes in response to ischemic-reperfusion injury in rats [41]. Additionally, elevated CSF haptoglobin levels have be seen in idiopathic normal pressure hydrocephalus, traumatic brain injury, Alzheimer's disease, multiple sclerosis, neuromyelitis optica, and Guillain-Barré syndrome [42], [43], [44], [45], [46], and therefore may not be specific to HD. In our study, CSF haptoglobin of HD patients was elevated but the serum haptoglobin was not when compared with the controls, which hints the increased intrathecal synthesis of haptoglobin in HD. However, the CSF haptoglobin was not correlated to the disease severity. Although the exact function of haptoglobin in HD is unclear, the elevated haptoglobin may represent the compensatory response to a serial inflammatory cascade in CNS.

Compared with the findings of proteome analyses in HD CSF published previously by Fang and colleagues where several neuronal proteins were found to be reduced [12], our results identified only three up-regulated protein molecules, which may, in part, due to that we did not deplete CSF abundant protein before 2-DE analysis, which could mask the small differences in low abundant protein and thus the sensitivity of proteomic techniques to identify proteins of biological interest could be reduced. However, many proteins, no matter large or small, are binding to or associated with high abundant protein such as albumin. Therefore, depletion of high abundant protein may also deplete some biologically important proteins. In this regard, we chose not to deplete the high abundant protein before 2-DE analysis [47]. Furthermore, our results are robust, because the three proteins were further confirmed to be elevated in HD CSF using western analysis or ELISA assay. Interestingly, the elevated Apo A-IV, prothrombin, and haptoglobin were speculated to be produced in central nervous system (CNS), based on intact BBB in HD patients shown for the first time by the present study, although it is possible that the increased proteins may be carried into CNS by their transporters. Intact BBB in HD also supports that mutant huntingtin induces parallel cell dysfunction in both CNS and peripheral system. Future studies of BBB integrity in HD mice are warranted to confirm our finding.

Our study results have provided insights into the pathogenesis of HD and identified a few potential biomarkers for HD. However, there are still some limitations. Our HD patients received variable medications including tetrabenazine, amantadine, ubidecarenone, neuroleptics (risperidone, haloperidol, and quetiapine), anticonvulsants (phenytoin, valproate and clonazepam), and antidepressants (paroxetine and mirtazapine). Although there were no previous studies exploring the interactions of medications to prothrombin, Apo A-IV or haptoglobin, the possibility that the medications could be a confounding variable can not be completely excluded. Since our study enrolled a small number of symptomatic patients, whether or not the results could be seen in pre-symptomatic HD carriers remains to be clarified. Like most of studies on CSF, it is difficult to obtain the CSF from healthy individuals due to the ethical issue, which may add a confounding factor to our results. In addition, there is no systematically comparison of the changes in CSF of HD with other diseases, and thus the specificity of the elevated protein in HD is not clear. Hence, further studies with a large series of cases including pre-symptomatic HD carriers are necessary to predict disease onset and disease progression of HD.
